# Association between cardiometabolic index and overactive bladder in adult American women: A cross-sectional study

**DOI:** 10.1371/journal.pone.0314594

**Published:** 2025-01-14

**Authors:** Junhua Li, Min He, Yu Zhou

**Affiliations:** 1 School of Mathematics and Computer Science, Hanjiang Normal University, Shiyan, Hubei, China; 2 Department of Urology, Renmin Hospital, Hubei University of Medicine, Shiyan, Hubei, China; 3 Department of Respiratory and Critical Care Medicine, Renmin Hospital, Hubei University of Medicine, Shiyan, Hubei, China; Universidad Autonoma de Chihuahua, MEXICO

## Abstract

**Background:**

Overactive bladder (OAB) is a common disorder, particularly in women, and its symptoms, including urgency, frequency, and nocturia, can significantly affect quality of life. The cardiometabolic index (CMI) is a novel metabolic risk indicator that has been receiving more attention lately. This study investigated the association between CMI and OAB in adult women.

**Methods:**

A cross-sectional analysis was performed using data from the National Health and Nutrition Examination Survey (NHANES) covering the years 2007 to 2018, including 6323 female participants. CMI was calculated based on waist-to-height ratio, triglyceride, and HDL cholesterol levels, while OAB was assessed using the overactive bladder symptom score (OABSS). The association between CMI and OAB was evaluated through multivariate logistic regression, generalized additive models (GAM), smoothing curve fitting, and subgroup analysis. We finally included male participants for sensitivity analysis.

**Results:**

A significant positive association was found between female CMI and OAB prevalence (OR = 1.46, 95% CI: 1.29–1.65). When compared to the lowest CMI quartile (Q1), women in the highest CMI quartile were 70% more likely to have OAB (OR = 1.70, 95% CI: 1.42–2.04). Smoothed curve fitting analysis showed a linear association between CMI and OAB. Subgroup analysis revealed that the association between CMI and OAB was stronger in women aged 20–50 years as well as in women without hypertension. Sensitivity analysis confirmed the robustness of our result.

**Conclusion:**

CMI was significantly and positively associated with the prevalence of OAB, especially in women aged 20–50 years without hypertension. This finding provides a new perspective on metabolic risk management and may contribute to the early prevention and improvement of bladder function in women.

## 1. Background

Patients with overactive bladder syndrome (OAB) may have significant reductions in their quality of life due to symptoms such as urgency, frequency, and nocturia [1, 2]. The European Prospective Investigation into Cancer and Nutrition (EPIC) research, which included 19,165 people from five European nations, discovered that 11.8% of participants had OAB, with a particularly high incidence in women [3]. In contrast to other lower urinary tract symptoms, OAB symptoms are more complex, affecting not only physical health but also having a detrimental effect that is considerable on the psychosocial well-being of patients [4]. Although the causes of OAB are multifactorial, the traditional view holds that it is primarily caused by the involuntary contraction of the detrusor muscle of the bladder [5]. Nevertheless, growing evidence suggests that disorders related to lipid metabolism may also be crucial in the onset and development of OAB [6]. In an epidemiological survey, Yu *et al*. demonstrated that hyperlipidemia is associated with the manifestation of OAB in Taiwanese women [7]. Additionally, a cross-sectional study showed that women with a body fat percentage of 32% or higher are more likely to develop OAB [8, 9].

The cardiometabolic index (CMI) is a comprehensive indicator of cardiovascular and metabolic health, integrating parameters such as waist circumference, height, triglycerides, and high-density lipoprotein cholesterol [10, 11]. CMI is not only widely used in assessing an individual’s risk of cardiovascular disease but is also considered sensitive to metabolic disorders such as insulin resistance and visceral fat accumulation [12, 13]. Previous research has shown that a number of metabolic disorders, such as diabetes, hypertension, and coronary heart disease, are strongly correlated with higher CMI readings [13–15]. These diseases themselves are associated with bladder dysfunction. For example, hyperglycemic states may lead to bladder nerve dysfunction, while obesity and increased visceral fat may impair normal bladder function through inflammatory pathways [16, 17]. Therefore, as a comprehensive metabolic risk assessment tool, CMI may indirectly or directly influence the development of overactive bladder by reflecting metabolic disorder status. In recent years, the cardiometabolic index (CMI), as a crucial measure of metabolic well-being, has garnered increasing attention in studies examining its relationship with various chronic diseases. Emerging evidence suggests that metabolic disorders not only impact cardiovascular health but may also affect lower urinary tract function, particularly bladder function [18].

Although some studies have shown an association between metabolic syndrome and OAB, few have explored the direct relationship between CMI and OAB. Most studies have focused on the impact of a single metabolic risk factor (such as obesity or diabetes) on OAB, overlooking the overall effect of metabolic disorders on bladder function. The aim is to uncover the potential link between CMI and OAB and give the metabolic management of OAB a theoretical foundation. The study’s findings will offer foundational data for further exploration of the association between metabolic health and bladder dysfunction, and present new perspectives for the early prevention and metabolic management of OAB.

## 2. Materials and methods

### 2.1 Data availability

The National Health and Nutrition Examination Survey (NHANES) is a long-term project run by the National Center for Health Statistics (NCHS) to assess the health and nutrition status of the U.S. population. Utilizing a multi-stage sampling method, NHANES provides a representative sample of nationwide health data, covering aspects such as socioeconomic status, lifestyle, and disease conditions. The survey collects comprehensive information on demographics, nutritional intake, health risks, and more through a combination of questionnaires, physical exams, home visits, and laboratory analyses. Data are collected biennially, made publicly available, and support a wide range of research and analytical purposes.

The portions of this study involving human participants, human materials, or human data were conducted in accordance with the Declaration of Helsinki and were approved by the National Center for Health Statistics (NCHS) Ethics Review Board. The patients/participants provided their written informed consent to participate in this study. Detailed information and data resources are accessible on the CDC’s official website: https://www.cdc.gov/nchs/nhanes/irba98.htm.

### 2.2 Study population

**Fig 1:**
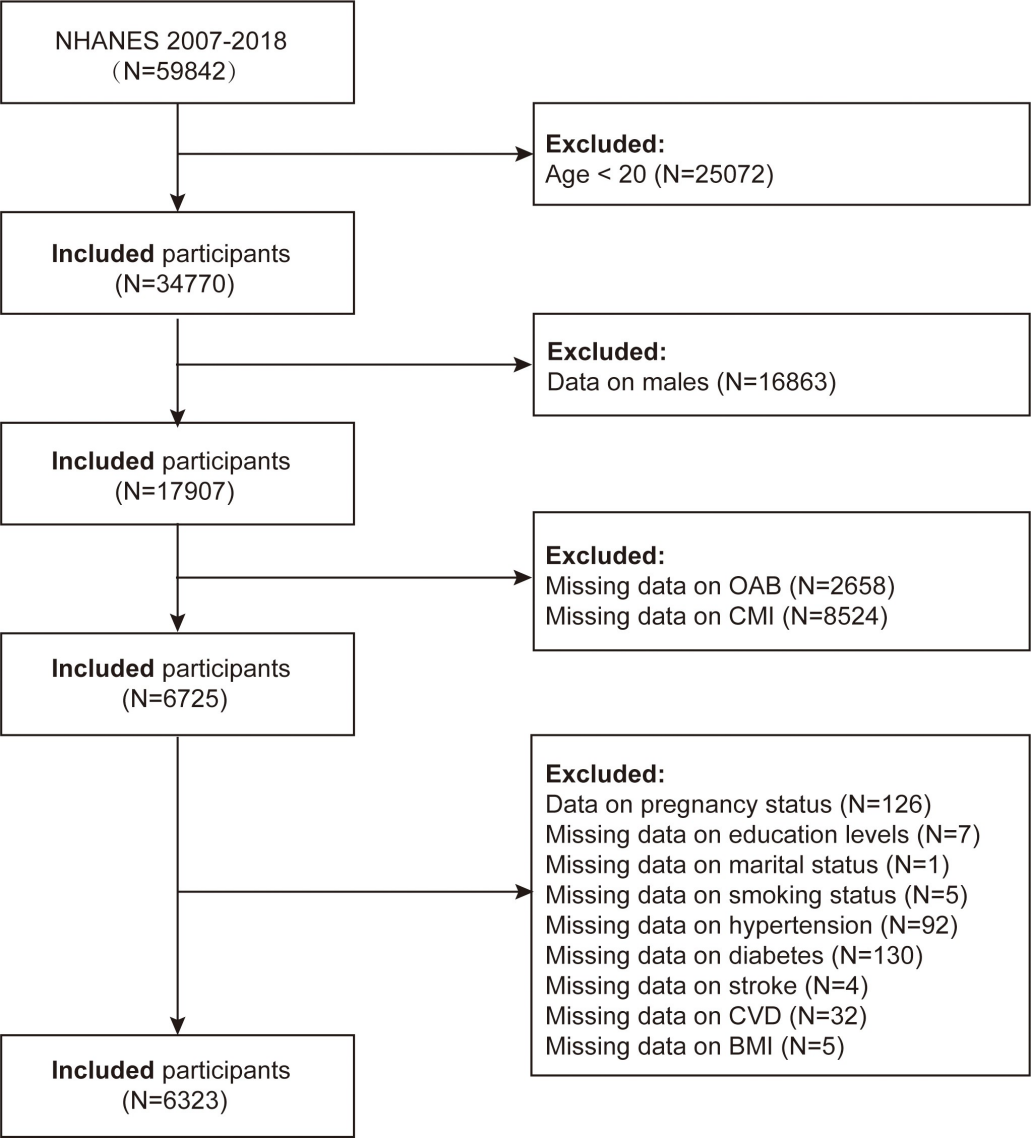
Flowchart of sample selection from NHANES 2007–2018.

In this study, we downloaded six consecutive datasets (2007–2008, 2009–2010, 2011–2012, 2013–2014, 2015–2016, and 2017–2018) from the NHANES database to precisely evaluate the relationship among CMI and OAB. Initially, 59,842 participants were included. The following were the exclusion standards: (1) individuals under the age of twenty (n = 25,072); (2) male participants (n = 16,863); (3) participants with missing OAB data (n = 2,658); (4) participants with missing CMI index data (n = 8,524); (5) participants with missing pregnancy status data (n = 126); (6) individuals for whom the education level data is lacking (n = 7); (7) individuals whose marital status information is missing (n = 1); (8) participants with missing smoking status data (n = 5); (9) participants with missing hypertension data (n = 92); (10) participants with missing diabetes data (n = 130); (11) participants with missing stroke data (n = 4); (12) individuals whose CVD data is missing (n = 32); and (13) individuals whose BMI data is missing (n = 5). Ultimately, the final analysis comprised 6,323 individuals. (Fig 1)

### 2.3 Assessment of cardiometabolic risk index

The CMI is calculated using anthropometric and biochemical data, including height, waist circumference (WC), triglycerides (TG), and high-density lipoprotein cholesterol (HDL-C). Height and waist circumference are measured in centimeters (cm), while triglycerides and HDL-C are measured in millimoles per liter (mmol/L). The CMI is calculated as follows [10, 19]:

$$\begin{array}{c} WHtR=\frac{WC}{height}\\ CMI=\frac{TG}{HDL-C}\times WHtR \end{array}$$

### 2.4 Assessment of OAB

OAB is a symptomatic syndrome characterized by urinary urgency, typically accompanied by frequent micturition and nocturia, with or without urge incontinence (UUI), and in the absence of urinary tract infection or other obvious pathology. We used the NHANES database’s urology questionnaire for kidney disease to assess OAB. The questions “Have you ever urinated before reaching the toilet?” and “How often does this happen?” were used to evaluate the severity of UUI, while “How many times do you urinate at night?” assessed the severity of nocturia. Based on previous research [23], we quantified the severity of OAB for each participant using the Overactive Bladder Symptom Score (OABSS) scale, with participants scoring ≥ 3 considered to have OAB [19, 20]. For the detailed scoring table, see **S1 Table.**

### 2.5 Covariate

In this study, covariates included age and race (Mexican American, other Hispanic, non-Hispanic White, non-Hispanic Black, and other races). Educational level was categorized as below high school, high school, and above high school. Marital status was classified as never married, married/living with a partner, and widowed/divorced/separated. The family income poverty ratio (PIR) was divided into three groups: <1.3 (low income), 1.3–3.5 (middle income), and >3.5 (high income). Body mass index (BMI) was categorized into three groups: <25 kg/m^2^, 25–30 kg/m^2^, and ≥30 kg/m^2^. Smoking status was classified into three categories: current smoker (has smoked 100 or more cigarettes and is still smoking), former smoker (has smoked 100 or more cigarettes but has quit), and never smoker (has smoked fewer than 100 cigarettes or never smoked). A drinker was defined as someone who drank more than 12 times in any given year during their lifetime. Diabetes was defined as a doctor’s diagnosis or a fasting blood glucose level of ≥126 mg/dL. Hypertension was defined as a previous diagnosis, current use of antihypertensive medication, or an average of three blood pressure readings of ≥140/90 mmHg. Stroke was categorized as yes or no. Cardiovascular disease (CVD) includes a history of congestive heart failure, coronary artery disease, angina, or heart attack.

### 2.6 Statistical analyses

This analysis followed the complex multi-stage sampling weight guidelines provided by NHANES. In this study, baseline information was divided into quartiles according to CMI. Continuous variables were expressed as weighted means with standard deviations (SD), while categorical variables were expressed as percentages. Analysis of variance (ANOVA) and chi-square tests were used to compare the distribution of baseline characteristics between groups.

This study constructed three weighted logistic regression models to explore the association between female CMI and OAB. Model 1 was unadjusted. Model 2 was adjusted for age and race. Model 3 was adjusted for education level, marital status, PIR, smoking, drinking, diabetes, hypertension, cardiovascular disease, and stroke, in addition to the covariates in Model 2. We performed weighted multiple regression analysis with CMI as both a continuous and categorical variable (quartiles) to describe the association between CMI and OAB and estimated the trend by treating the CMI quartiles as a continuous variable. Additionally, we further analyzed the non-linear association between CMI and OAB prevalence using generalized additive models (GAM) and smoothing curve fitting. Subgroup analyses and interaction tests were then conducted for potential confounders listed in the baseline table. We then reincluded male participants to perform a multivariate logistic regression analysis between CMI and OAB as part of the sensitivity analysis. Statistical analyses were conducted using R (4.3.3 version) and EmpowerStats (4.20 version), with the significance level set at P < 0.05.

## 3. Results

### 3.1 Weighted baseline characteristics of the study population

The study included 6,323 female participants with a weighted mean age of 49.02 ± 17.03 years and a mean CMI of 0.62 ± 0.87. The overall prevalence of OAB among participants was 27.27%, showing a significant increase across rising CMI quartiles (P < 0.05). Individuals in the higher CMI quartiles were more likely to be Mexican American, have a PIR < 1.3, have lower education levels, BMI ≥ 30, and be smokers. They also demonstrated a higher prevalence of hypertension, diabetes, coronary heart disease, and stroke compared to participants with lower CMI values (P < 0.05) **(Table 1).**

**Table 1:** Baseline characteristics of the study population, weighted according to CMI quartiles.

Characteristics	Total (n = 6323)	CMI Quartile				*P* value
		Q1 (n = 1581)	Q2 (n = 1580)	Q3 (n = 1581)	Q4 (n = 1581)	
Age (years, mean ± SD)	49.02 ± 17.03	44.88 ± 16.97	48.89 ± 17.42	49.87 ± 17.03	53.05 ± 15.55	<0.0001
Race (%)						<0.0001
Mexican American	7.84	5.20	7.29	9.18	10.06	
Other Hispanic	5.64	4.96	5.29	6.12	6.31	
Non-Hispanic White	68.19	66.87	69.64	65.56	70.88	
Non-Hispanic Black	10.95	13.47	12.15	11.82	5.86	
Other Race	7.39	9.50	5.64	7.32	6.89	
Education level (%)						<0.0001
Less than high school	14.71	7.35	12.91	17.19	22.52	
High school	22.65	15.77	23.82	24.46	27.46	
More than high school	62.64	76.88	63.27	58.35	50.02	
Marital status (%)						<0.0001
Never married	15.95	19.92	16.10	16.18	11.01	
Married/Living with partner	60.76	62.38	58.86	59.85	61.87	
Widowed/divorced/Separated	23.28	17.70	25.05	23.97	27.12	
PIR (%)						<0.0001
<1.3	23.17	18.19	22.47	23.67	29.11	
1.3–3.5	36.03	32.54	33.86	40.51	37.70	
≥3.5	40.81	49.27	43.68	35.82	33.18	
BMI (%)						<0.0001
<25	33.14	64.62	34.74	21.53	7.26	
25–30	28.27	23.22	33.94	31.12	25.13	
≥30	38.58	12.16	31.32	47.35	67.62	
Smoking status (%)						<0.0001
Never	61.39	68.49	61.84	61.67	52.47	
Now	17.44	11.75	17.19	19.45	22.19	
Former	21.16	19.76	20.97	18.89	25.35	
Alcohol intake (%)						0.0009
No	11.70	9.42	12.40	11.37	13.91	
Yes	88.30	90.58	87.60	88.63	86.09	
Hypertension (%)						<0.0001
No	61.76	77.09	65.10	56.81	45.71	
Yes	38.24	22.91	34.90	43.19	54.29	
Diabetes (%)						<0.0001
No	86.45	97.04	91.38	83.57	72.01	
Yes	13.55	2.96	8.62	16.43	27.99	
Stroke (%)						0.0153
No	96.72	97.84	95.98	96.67	96.28	
Yes	3.28	2.16	4.02	3.33	3.72	
CVD (%)						<0.0001
No	94.08	97.01	94.75	93.66	90.45	
Yes	5.92	2.99	5.25	6.34	9.55	
TC (mmol/L, mean ± SD)	5.08 ± 1.07	4.87 ± 0.95	4.98 ± 0.99	5.17 ± 1.16	5.34 ± 1.12	<0.0001
HDL-C (mmol/L, mean ± SD)	1.54 ± 0.44	1.91 ± 0.39	1.60 ± 0.31	1.42 ± 0.42	1.18 ± 0.22	<0.0001
TG (mmol/L, mean ± SD)	1.28 ± 1.04	0.65 ± 0.19	0.97 ± 0.24	1.48 ± 1.61	2.13 ± 0.68	<0.0001
Height (cm, mean ± SD)	161.99 ± 6.90	163.06 ± 6.81	162.06 ± 6.96	161.73 ± 6.82	160.95 ± 6.85	<0.0001
Waist circumference (cm, mean ± SD)	97.15 ± 16.87	84.92 ± 11.46	95.07 ± 14.27	100.66 ± 15.88	109.80 ± 15.21	<0.0001
WHtR (mean ± SD)	0.60 ± 0.11	0.52 ± 0.07	0.59 ± 0.09	0.62 ± 0.10	0.68 ± 0.09	<0.0001
CMI (mean ± SD)	0.62 ± 0.87	0.18 ± 0.05	0.35 ± 0.05	0.77 ± 1.48	1.26 ± 0.47	<0.0001
OAB (%)						<0.0001
No	72.73	81.68	76.45	70.29	61.00	
Yes	27.27	18.32	23.55	29.71	39.00	

Continuous variables are expressed as mean and standard deviation (SD) and categorical variables are expressed as percentages.

Abbreviations: PIR, poverty income ratio; BMI, body mass index; HDL-C, high-density lipoprotein cholesterol; TG, triglyceride; TC, total cholesterol; CMI, cardiometabolic index; WHtR, waist-to-height ratio; WC, waist circumference; CVD, cardiovascular disease.

We have included a supplementary table **(S4 Table**) to present a comparative overview of the baseline characteristics between the OAB and non-OAB groups. The results indicate that the CMI value in the OAB group was significantly higher than in the non-OAB group (mean CMI: 0.77 ± 1.11, P<0.05). Additionally, the OAB group showed a notably higher proportion of individuals with advanced age, higher BMI, smoking and alcohol consumption, and comorbidities such as hypertension, diabetes, CVD, and history of stroke; all of these factors exhibited significant differences between the groups (P<0.05).

### 3.2 The association between CMI and female OAB

**Fig 2:**
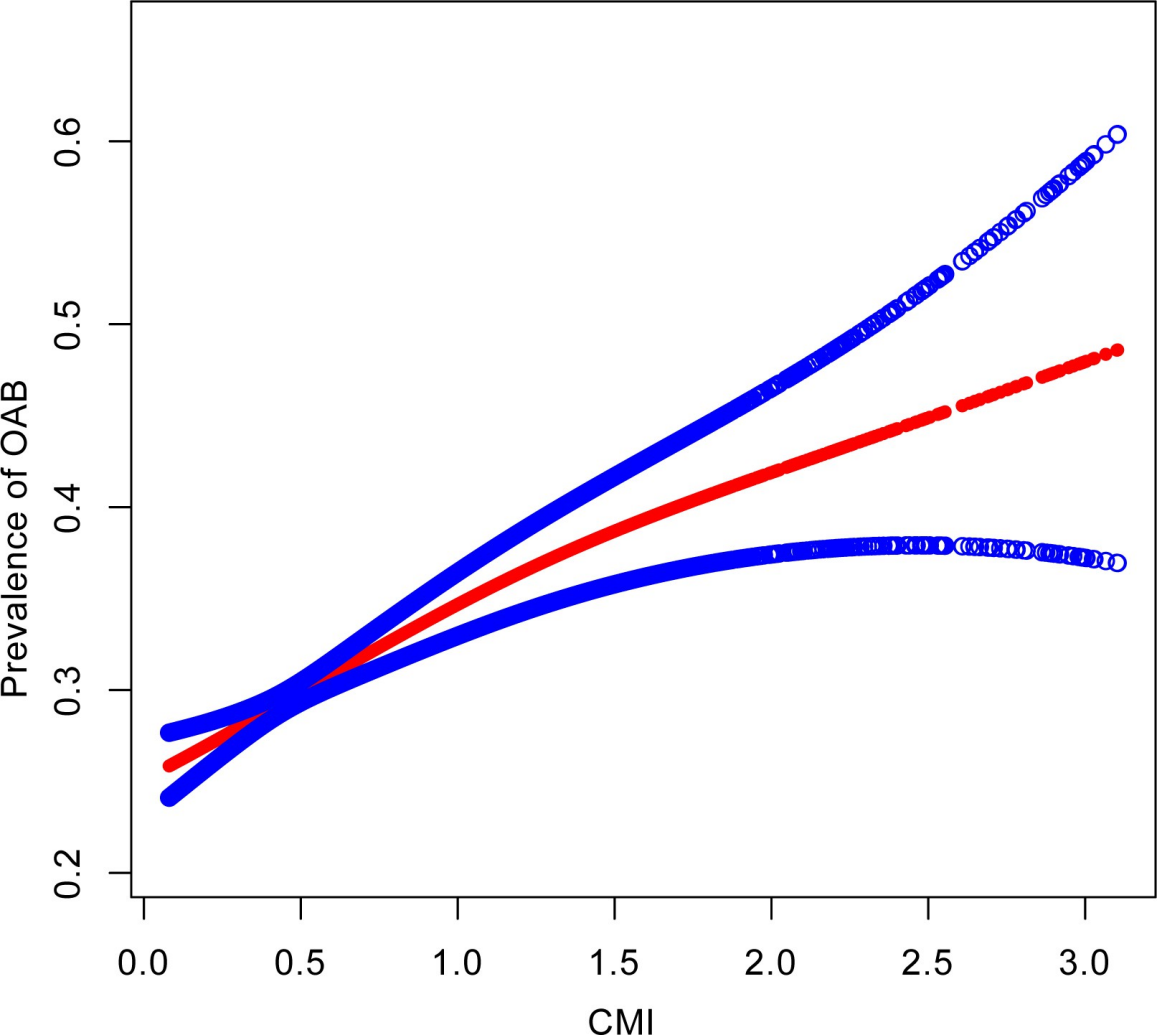
Dose-response relationship between CMI and female OAB prevalence. The associations were adjusted for age, race, education level, marital status, PIR, smoking status, alcohol consumption, diabetes, hypertension, CVD, and stroke.

The results of the weighted multiple logistic regression indicated that CMI was significantly and positively associated with OAB in both the unadjusted model (OR = 1.94, 95% CI: 1.74, 2.16) and the fully adjusted model (OR = 1.46, 95% CI: 1.29, 1.65). Additionally, we transformed CMI from a continuous to a categorical variable (quartiles). In Model 3, compared to participants in the lowest CMI quartile (Q1), those in the highest CMI quartile had a 70% increased likelihood of having OAB (OR = 1.70, 95% CI: 1.42, 2.04). A significant trend was observed across the CMI quartiles (p for trend < 0.001) **(Table 2).** Additionally, generalized additive models and smooth curve fitting showed a linear relationship between CMI and OAB (log-likelihood ratio test p-value > 0.05). **(Fig 2) (Table 3)**

**Table 2:** Weighted multiple logistic regression analysis of the association between CMI and female OAB prevalence.

Characteristic	Model 1		Model 2		Model 3	
	OR (95% CI)	*P*-value	OR (95% CI)	*P*-value	OR (95% CI)	*P*-value
CMI (continuous)	1.94 (1.74, 2.16)	<0.001	1.77 (1.58, 1.99)	<0.001	1.46 (1.29, 1.65)	<0.001
CMI (quartile)						
Q1	1.0 (ref)		1.0 (ref)		1.0 (ref)	
Q2	1.52 (1.29, 1.80)	<0.001	1.27 (1.06, 1.51)	0.008	1.17 (0.98, 1.39)	0.089
Q3	1.93 (1.64, 2.27)	<0.001	1.53 (1.29, 1.82)	<0.001	1.30 (1.09, 1.55)	0.003
Q4	2.85 (2.43, 3.34)	<0.001	2.21 (1.86, 2.63)	<0.001	1.70 (1.42, 2.04)	<0.001
P for trend		<0.001		<0.001		<0.001

Model 1: No covariates were adjusted.

Model 2: Adjusted for age and race.

Model 3: Adjusted for age, race, education level, marital status, PIR, smoking status, alcohol consumption, diabetes, hypertension, CVD, and stroke.

**Table 3:** Threshold effect analysis of the association between CMI and female OAB prevalence.

Outcome: OAB	Adjusted OR (95% CI)	*P*-value
Fitting by standard linear model	1.46 (1.29, 1.65)	<0.001
Fitting by two-piecewise linear model		
Inflection point	1.21	
OR1< 1.21	1.73 (1.40, 2.14)	<0.001
OR2> 1.21	1.16 (0.89, 1.51)	0.284
Logarithmic likelihood ratio test *P*-value		0.054

The associations were adjusted for age, race, education level, marital status, PIR, smoking status, alcohol consumption, diabetes, hypertension, CVD, and stroke.

### 3.3 Subgroup analyses

**Fig 3:**
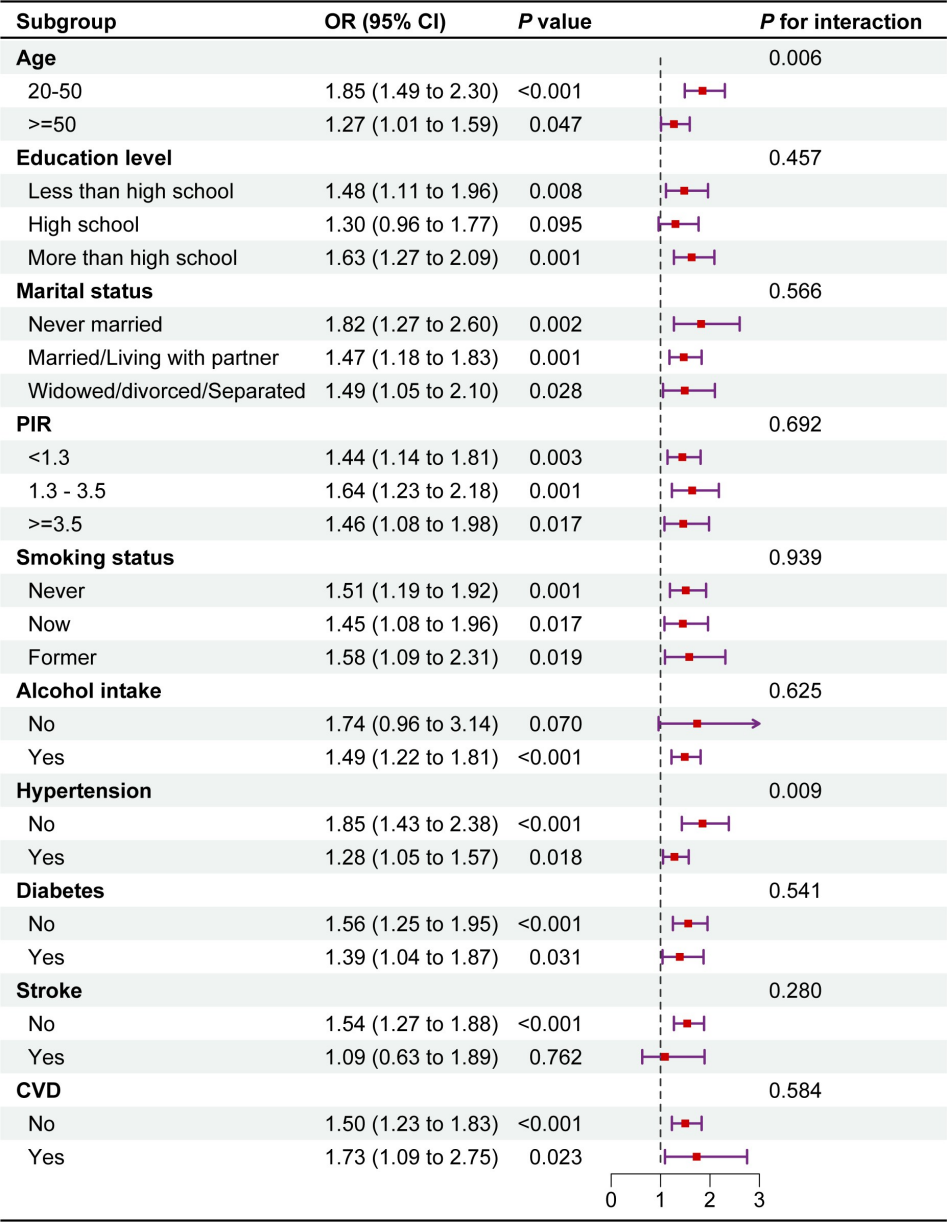
Subgroup analysis of the association between CMI and female OAB prevalence. Note 1: The above model adjusted for age, race, education level, marital status, PIR, smoking status, alcohol consumption, diabetes, hypertension, CVD, and stroke. Note 2: In each case, the model was not adjusted for the stratification variable.

The results showed that CMI was positively associated with the prevalence of female OAB in all subgroups. Interestingly, the association between CMI and female OAB prevalence varied by age and hypertension subgroups (P for interaction < 0.05). The connection was more pronounced in the non-hypertensive population (OR = 1.85, 95% CI: 1.43, 2.83) and in the 20–50 age range (OR = 1.85, 95% CI: 1.49, 2.30) **(Fig 3).**

Subgroup analyses based on CMI quartiles showed a trend towards increased positive correlation between CMI and OAB across subgroups in Q4 compared to the lowest CMI quartile (Q1). (**S3 Table**)

### 3.4 Sensitivity analysis

**Fig 4:**
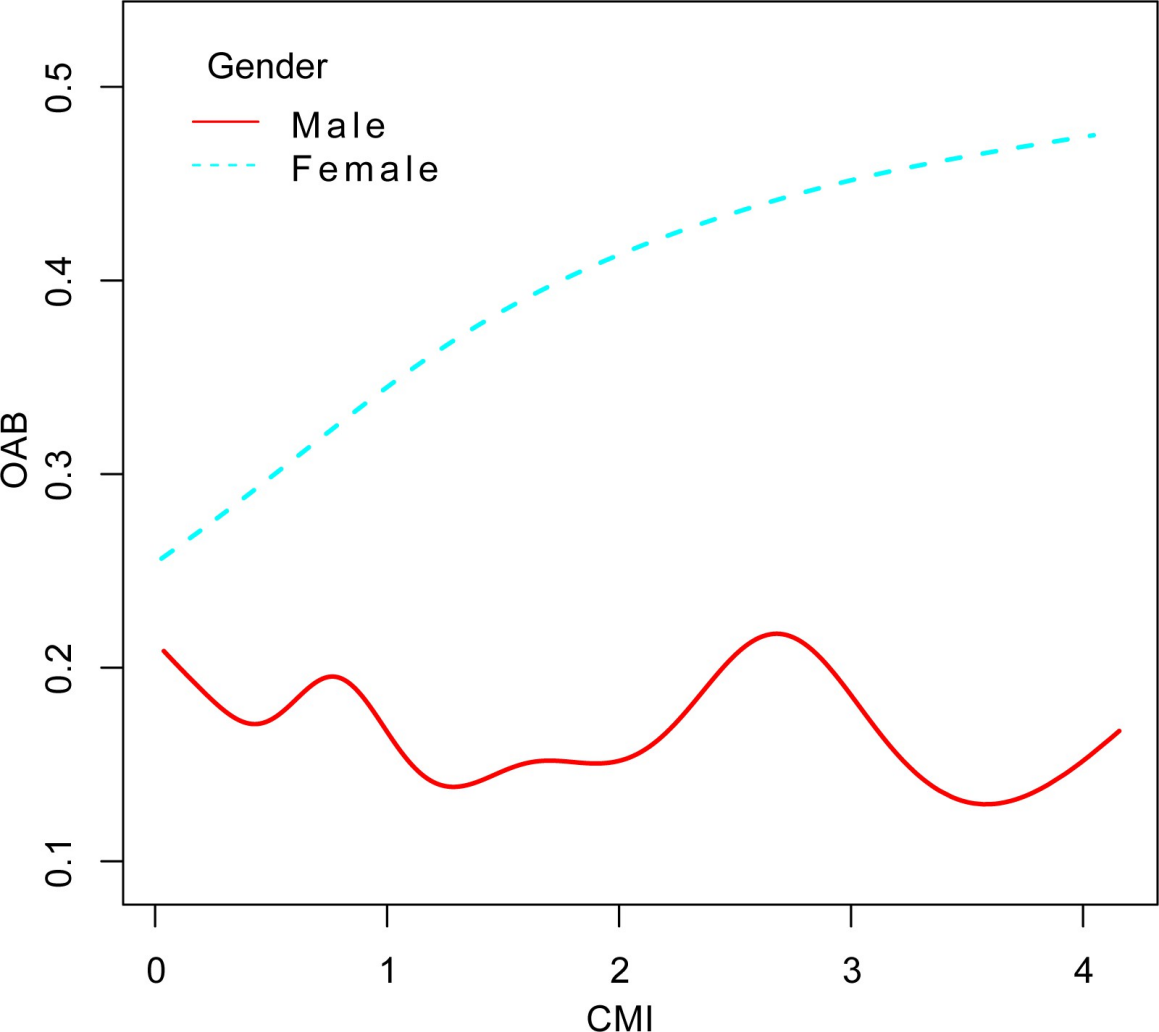
Smooth curve fitting analysis based on gender stratification. Adjusted for age, race, education level, marital status, PIR, smoking status, alcohol consumption, diabetes, hypertension, CVD, and stroke.

We included male participants for sensitivity analysis **(S1 Fig and S2 Table)**. In Model 3, multivariate logistic regression results demonstrated a persistent positive association between CMI and OAB (OR = 1.07, 95% CI: 1.03, 1.12). Gender-stratified analysis and smooth curve fitting analysis suggested that the association between CMI and OAB may be stronger in women (OR = 1.13, 95% CI: 1.05, 1.22) (**Fig 4 and Table 4**).

**Table 4:** Multivariate logistic regression analysis including both male and female participants.

Characteristic	Model 1		Model 2		Model 3	
	OR (95% CI)	*P*-value	OR (95% CI)	*P*-value	OR (95% CI)	*P-*value
CMI (continuous)	1.08 (1.05, 1.12)	<0.001	1.15 (1.11, 1.20)	<0.001	1.07 (1.03, 1.12)	<0.001
CMI (quartile)						
Q1	1.0 (ref)		1.0 (ref)		1.0 (ref)	
Q2	1.25 (1.11, 1.41)	<0.001	1.14 (1.00, 1.30)	0.049	1.04 (0.91, 1.19)	0.535
Q3	1.48 (1.31, 1.66)	<0.001	1.39 (1.22, 1.58)	<0.001	1.16 (1.01, 1.33)	0.029
Q4	1.60 (1.42, 1.80)	<0.001	1.73 (1.52, 1.97)	<0.001	1.26 (1.10, 1.45)	<0.001
P for trend		<0.001		<0.001		<0.001
Gender grouping						
CMI (male)	1.06 (1.02, 1.11)	0.0071	1.11 (1.06, 1.17)	<0.001	1.07 (1.02, 1.12)	0.008
CMI (female)	1.39 (1.28, 1.51)	<0.001	1.34 (1.23, 1.46)	<0.001	1.13 (1.05, 1.22)	0.002

Model 1: No covariates were adjusted.

Model 2: Adjusted for gender, age and race.

Model 3: Adjusted for gender, age, race, education level, marital status, PIR, smoking status, alcohol consumption, diabetes, hypertension, CVD, and stroke.

## 4. Discussion

This study is the first to examine the relationship between CMI and OAB in an adult female population in the United States. Our findings revealed a strong positive association between OAB prevalence and CMI, with the highest risk observed in the uppermost CMI quartile (Q4). This highlights that metabolic disorders, especially the overall metabolic health status as measured by CMI, may be key risk factors for OAB. Furthermore, the association was notably stronger in non-hypertensive and younger women, suggesting that specific metabolic profiles may have a more substantial impact on bladder health in these subgroups. Sensitivity analysis indicates that the positive association between CMI and OAB is stronger in women compared to men.

Even while previous research has established a link between OAB and certain metabolic risk factors such as diabetes, hypertension, and obesity, few have employed CMI, a comprehensive metabolic index, to assess the impact of metabolic disorders on OAB. Compared with previous studies that only focused on a single metabolic factor, our study provides a more comprehensive perspective and reveals the potential link between overall metabolic health and OAB. Obesity, in particular, has been linked to OAB symptoms in various populations. For example, according to one study, kids who are obese are more likely than kids who are average weight to have OAB symptoms. An increased body roundness index (BRI) was significantly correlated with a higher risk of OAB in adults, according to a cross-sectional study, indicating that BRI could be a helpful tool for diagnosing OAB in clinical settings [21]. Furthermore, case-control research revealed notable variations in LUTS, such as voiding and storage symptoms, between obese and non-obese patients [22]. The body weight-adjusted waist circumference index is positively connected with the risk of OAB in adult American women, according to research by Yilei Shang et al [23]. Similarly, measurement of CMI can offer a more thorough comprehension of the risk of hypertension associated with changes in body fat distribution and identify hypertensive participants at high risk of future cardiovascular disease [24]. Research by Ichiro Wakabayashi et al. indicated that CMI is a useful new indicator that reflects both obesity and blood lipids and can be used to distinguish individuals with diabetes [25]. Additionally, research has demonstrated a substantial correlation between a greater CMI and a higher likelihood of the metabolically obese normal weight (MONW) phenotype in Chinese adults. CMI is a useful technique for identifying the MONW phenotype in routine health checks because of its high sensitivity and excellent discriminative capabilities, especially for young people [26]. These findings are consistent with our results, highlighting the importance of CMI as a comprehensive metabolic indicator in assessing the risk of OAB.

Studies have shown that women with a high CMI are more likely to develop OAB, which may be closely related to the multifaceted effects of metabolic disorders on bladder function [6]. In particular, core components of CMI, such as increased waist circumference and elevated triglyceride levels, are associated with insulin resistance, increased visceral fat, and chronic low-grade inflammation [16, 17]. These metabolic disorders not only affect the cardiovascular system but may also influence urinary system function through various mechanisms. Specifically, increased waist circumference reflects the accumulation of visceral fat, which, as a metabolically active tissue, releases numerous pro-inflammatory factors, such as tumor necrosis factor-α (TNF-α) and interleukin-6 (IL-6). These factors may exacerbate detrusor muscle excitability via systemic inflammatory pathways, thereby increasing the risk of OAB [6, 17, 27, 28]. Furthermore, Qingliu He et al.’s research indicates that systemic inflammation plays a significant role as a mediator between OAB and diabetes-related indicators, guiding further studies on OAB function and mechanisms [16]. Secondly, elevated triglycerides and reduced HDL-C are closely associated with endothelial dysfunction [29]. The bladder, being a highly vascularized organ, relies on adequate blood supply to maintain normal function. When endothelial function is impaired, reduced blood flow to the bladder may lead to detrusor muscle hypoxia, triggering abnormal detrusor contractions—a phenomenon confirmed in both animal models and human studies [30]. Therefore, the metabolic disorders reflected by CMI, such as elevated triglycerides and reduced HDL-C, may indirectly exacerbate OAB by affecting vascular function. Our findings support that the association between CMI and OAB may stem from these multifaceted effects on bladder function. Moreover, these metabolic abnormalities are known risk factors for cardiovascular disease, which may also impair bladder endothelial function, leading to increased bladder excitability [31]. The documented association between cardiovascular diseases, such as atherosclerosis, and bladder dysfunction further highlights the importance of vascular health in maintaining bladder function [31].

## 5. Strengths and limitations

This study has several important strengths, making it uniquely valuable and contributing to the existing literature. Firstly, our study utilizes data from NHANES (2007–2018), which includes a large, nationally representative sample. This ensures that our findings have high external validity and can more accurately reflect the association between CMI and OAB among adult women in the United States. Additionally, the NHANES data were collected using a rigorous multi-stage sampling design and data collection process, ensuring high data quality. While previous studies have explored the effects of individual metabolic risk factors (e.g., obesity, diabetes) on OAB, our study is the first to analyze the relationship between CMI, a comprehensive metabolic index, and OAB. CMI incorporates multiple metabolic parameters (waist circumference, triglycerides, and HDL-C), providing a more comprehensive reflection of an individual’s metabolic health. Therefore, this study offers a more integrated perspective on the association between metabolic health and bladder dysfunction.

This work has some drawbacks, despite its significant contributions. First, this study’s cross-sectional approach implies that we can only observe the association between CMI and OAB at a specific point in time and cannot infer causality. Therefore, although we found a significant association between CMI and OAB, we cannot determine whether metabolic health problems are a direct cause of OAB or whether OAB in turn aggravates metabolic disorders. Secondly, even though we conducted a study that took several possible confounding factors into account, unmeasured or residual confounders may still be present. Further longitudinal research is required to address these potential confounders and confirm the causal nature of this correlation.

## 6. Conclusion

The results suggest a positive association between CMI and OAB prevalence, particularly in women under 50 years of age. This study offers novel insights into the relationship between metabolic health and OAB, providing a theoretical foundation for targeted public health interventions and enhanced clinical management strategies.

## Supporting information

S1 Table Criteria for conversion of symptom frequencies recorded in NHANES and OABSS scores. (DOCX)

S2 Table Weighted baseline table of the study population for male and female participants. (DOCX)

S3 Table Subgroup analysis based on CMI quartiles. (DOCX)

S4 Table Baseline table of participant population based on OAB subgroups. (DOCX)

S1 Fig A flowchart of the study population, including male and female participants. (TIF)

S1 Data Data used for analysis in the study. (XLSX)

## Abbreviations

NHANES: National Health and Nutrition Examination Survey
OAB: Overactive bladder
CMI: cardiometabolic index
GAM: generalized additive model
OABSS: overactive bladder symptom score
PIR: income to poverty ratio
CVD: cardiovascular disease
BMI: body mass index
NCHS: National Center for Health Statistics
WHtR: waist-to-height ratio
WC: waist circumference
HDL-C: high-density lipoprotein cholesterol
TG: triglycerides
LUTS: lower urinary tract symptoms
UUI: urgency urinary incontinence
