# Supplementary material for: Association between cardiometabolic index and overactive bladder in adult American women: A cross-sectional study
Source: PLoS One. 2025 Jan 14;20(1):e0314594. doi: 10.1371/journal.pone.0314594 (PMC11731727; doi:10.1371/journal.pone.0314594)
Supplement: S1 Table — (DOCX) [file pone.0314594.s001.docx]

**Table S1. Criteria for Conversion of Symptom Frequencies recorded in NHANES and OABSS Scores.**

| **According to NHANES Score** | **According to OABSS Score** |
| --- | --- |
| Urge urinary incontinence frequency | Urge urinary incontinence score |
| Never | 0 |
| Less than once a month | 1 |
| A few times a month | 1 |
| A few times a week | 2 |
| Every day or night | 3 |
| Nocturia frequency | Nocturia score |
| 0 | 0 |
| 1 | 1 |
| 2 | 2 |
| 3 | 3 |
| 4 | 3 |
| 5 or more | 3 |
| When total score ≥3, the diagnosis is OAB | |

NHANES = National Health and Nutrition Examination Survey; OABSS = Overactive Bladder Symptom Score;
