# Supplementary material for: Association between cardiometabolic index and overactive bladder in adult American women: A cross-sectional study
Source: PLoS One. 2025 Jan 14;20(1):e0314594. doi: 10.1371/journal.pone.0314594 (PMC11731727; doi:10.1371/journal.pone.0314594)
Supplement: S2 Table — (DOCX) [file pone.0314594.s002.docx]

**Table S2.** Weighted baseline table of the study population for male and female participants.

| **Characteristics** | **Total (n = 12652)** | **Non-OAB (n = 9616)** | **OAB (n = 3036)** | ***P*-value** |
| --- | --- | --- | --- | --- |
| Age (years, mean ± SD) | 48.02 ±16.78 | 45.59 ±16.27 | 57.63 ±15.25 | <0.0001 |
| Gender (%) |  |  |  | <0.0001 |
| Male | 50.03 | 54.45 | 32.57 |  |
| Female | 49.97 | 45.55 | 67.43 |  |
| Race (%) |  |  |  | <0.0001 |
| Mexican American | 8.58 | 8.89 | 7.34 |  |
| Other Hispanic | 5.84 | 6.07 | 4.93 |  |
| Non-Hispanic White | 68.23 | 68.00 | 69.11 |  |
| Non-Hispanic Black | 10.05 | 9.27 | 13.15 |  |
| Other Race | 7.3 | 7.77 | 5.46 |  |
| Education level (%) |  |  |  | <0.0001 |
| Less than high school | 15.74 | 14.12 | 22.17 |  |
| High school | 22.99 | 22.31 | 25.68 |  |
| More than high school | 61.26 | 63.57 | 52.16 |  |
| Marital status (%) |  |  |  | <0.0001 |
| Never married | 17.68 | 19.66 | 9.83 |  |
| Married/Living with partner | 64.15 | 64.67 | 62.11 |  |
| Widowed/divorced/Separated | 18.17 | 15.67 | 28.06 |  |
| PIR (%) |  |  |  | <0.0001 |
| <1.3 | 21.85 | 20.61 | 26.75 |  |
| 1.3 - 3.5 | 35.29 | 34.48 | 38.47 |  |
| ≥3.5 | 42.86 | 44.91 | 34.77 |  |
| BMI (%) |  |  |  | <0.0001 |
| <25 | 29.77 | 31.89 | 21.38 |  |
| 25 - 30 | 33.21 | 34.02 | 30.04 |  |
| ≥30 | 37.02 | 34.09 | 48.58 |  |
| Smoking status (%) |  |  |  | <0.0001 |
| Never | 54.72 | 55.8 | 50.44 |  |
| Now | 19.37 | 19.09 | 20.48 |  |
| Former | 25.92 | 25.11 | 29.08 |  |
| Alcohol intake (%) |  |  |  | <0.0001 |
| No | 9.92 | 9.01 | 13.54 |  |
| Yes | 90.08 | 90.99 | 86.46 |  |
| Hypertension (%) |  |  |  | <0.0001 |
| No | 61.38 | 66.4 | 41.53 |  |
| Yes | 38.62 | 33.6 | 58.47 |  |
| Diabetes (%) |  |  |  | <0.0001 |
| No | 85.33 | 88.5 | 72.8 |  |
| Yes | 14.67 | 11.5 | 27.2 |  |
| Stroke (%) |  |  |  | <0.0001 |
| No | 97.15 | 98.1 | 93.39 |  |
| Yes | 2.85 | 1.9 | 6.61 |  |
| CVD (%) |  |  |  | <0.0001 |
| No | 92.85 | 94.89 | 84.80 |  |
| Yes | 7.15 | 5.11 | 15.2 |  |
| Height (cm) | 168.95 ±9.99 | 169.86 ±9.88 | 165.32 ±9.56 | <0.0001 |
| Waist circumference (cm, mean ± SD) | 99.49 ±16.49 | 98.23 ±16.05 | 104.48 ±17.28 | <0.0001 |
| HDL-C (mmol/L, mean ± SD) | 1.40 ±0.42 | 1.39 ±0.42 | 1.42 ±0.43 | 0.0202 |
| TG (mmol/L, mean ± SD) | 1.40 ±1.20 | 1.38 ±1.17 | 1.48 ±1.30 | 0.0001 |
| CMI (mean ± SD) | 0.75 ±1.04 | 0.72 ±0.96 | 0.84 ±1.30 | <0.0001 |

Continuous variables are expressed as mean and standard deviation (SD) and categorical variables are expressed as percentages.

Abbreviations: PIR, poverty income ratio; BMI, body mass index; HDL-C, high-density lipoprotein cholesterol; TG, triglyceride; CMI, cardiometabolic index; WC, waist circumference; CVD, cardiovascular disease.
