# Supplementary material for: Association between cardiometabolic index and overactive bladder in adult American women: A cross-sectional study
Source: PLoS One. 2025 Jan 14;20(1):e0314594. doi: 10.1371/journal.pone.0314594 (PMC11731727; doi:10.1371/journal.pone.0314594)
Supplement: S3 Table — (DOCX) [file pone.0314594.s003.docx]

**Table S3.** Subgroup analysis based on CMI quartiles.

| **Subgroup** | **CMI quartiles, OR（95%CI）*P*-value** | | | | ***P* for interaction** |
| --- | --- | --- | --- | --- | --- |
|  | **Q1** | **Q2** | **Q3** | **Q4** |  |
| **Age** |  |  |  |  | **0.0084** |
| 20-50 | 1.0 (ref) | 1.30 (0.91, 1.86) 0.1493 | 1.85 (1.32, 2.59) 0.0006 | 2.41 (1.75, 3.31) <0.0001 |  |
| ≥50 | 1.0 (ref) | 0.87 (0.64, 1.20) 0.4059 | 1.03 (0.75, 1.42) 0.8465 | 1.28 (0.93, 1.76) 0.1315 |  |
| **Education level** |  |  |  |  | 0.9354 |
| Less than high school | 1.0 (ref) | 0.94 (0.58, 1.51) 0.7856 | 1.29 (0.79, 2.11) 0.3047 | 1.58 (0.99, 2.54) 0.0605 |  |
| High school | 1.0 (ref) | 0.90 (0.56, 1.43) 0.6449 | 1.18 (0.76, 1.83) 0.4541 | 1.31 (0.78, 2.19) 0.3079 |  |
| More than high school | 1.0 (ref) | 1.11 (0.84, 1.47) 0.4566 | 1.35 (0.96, 1.89) 0.0862 | 1.86 (1.35, 2.56) 0.0003 |  |
| **Marital status** |  |  |  |  | 0.5081 |
| Never married | 1.0 (ref) | 1.69 (1.09, 2.63) 0.0223 | 1.84 (1.02, 3.31) 0.0464 | 2.74 (1.58, 4.77) 0.0007 |  |
| Married/Living with partner | 1.0 (ref) | 0.99 (0.73, 1.33) 0.9218 | 1.23 (0.90, 1.70) 0.2031 | 1.51 (1.12, 2.04) 0.0092 |  |
| Widowed/divorced/Separated | 1.0 (ref) | 0.98 (0.62, 1.54) 0.9244 | 1.34 (0.88, 2.04) 0.1754 | 1.72 (1.08, 2.73) 0.0250 |  |
| **PIR** |  |  |  |  | 0.4869 |
| <1.3 | 1.0 (ref) | 1.08 (0.74, 1.58) 0.6771 | 1.10 (0.79, 1.54) 0.5589 | 1.51 (1.04, 2.20) 0.0336 |  |
| 1.3 - 3.5 | 1.0 (ref) | 1.28 (0.92, 1.79) 0.1474 | 1.46 (1.02, 2.10) 0.0412 | 1.91 (1.34, 2.73) 0.0007 |  |
| ≥3.5 | 1.0 (ref) | 0.85 (0.57, 1.25) 0.4106 | 1.38 (0.86, 2.20) 0.1814 | 1.59 (1.08, 2.33) 0.0205 |  |
| **Smoking status** |  |  |  |  | 0.9810 |
| Never | 1.0 (ref) | 1.07 (0.83, 1.38) 0.6144 | 1.36 (0.98, 1.88) 0.0720 | 1.73 (1.24, 2.41) 0.0021 |  |
| Now | 1.0 (ref) | 1.14 (0.69, 1.88) 0.6198 | 1.44 (0.89, 2.33) 0.1446 | 1.58 (0.98, 2.56) 0.0666 |  |
| Former | 1.0 (ref) | 0.92 (0.59, 1.42) 0.7024 | 1.15 (0.71, 1.86) 0.5799 | 1.63 (1.03, 2.58) 0.0395 |  |
| **Alcohol intake** |  |  |  |  | 0.7389 |
| No | 1.0 (ref) | 1.10 (0.53, 2.29) 0.7934 | 1.04 (0.47, 2.30) 0.9306 | 1.57 (0.71, 3.51) 0.2698 |  |
| Yes | 1.0 (ref) | 1.03 (0.82, 1.29) 0.7891 | 1.37 (1.06, 1.77) 0.0198 | 1.69 (1.29, 2.20) 0.0003 |  |
| **Hypertension** |  |  |  |  | **0.0025** |
| No | 1.0 (ref) | 1.13 (0.83, 1.53) 0.4417 | 1.79 (1.31, 2.44) 0.0005 | 2.10 (1.50, 2.93) <0.0001 |  |
| Yes | 1.0 (ref) | 0.87 (0.63, 1.21) 0.4221 | 0.88 (0.64, 1.23) 0.4640 | 1.21 (0.89, 1.65) 0.2247 |  |
| **Diabetes** |  |  |  |  | 0.7111 |
| No | 1.0 (ref) | 1.02 (0.82, 1.28) 0.8550 | 1.34 (1.03, 1.73) 0.0304 | 1.61 (1.21, 2.14) 0.0018 |  |
| Yes | 1.0 (ref) | 1.42 (0.68, 2.99) 0.3534 | 1.53 (0.73, 3.20) 0.2620 | 2.27 (1.19, 4.33) 0.0155 |  |
| **Stroke** |  |  |  |  | 0.2909 |
| No | 1.0 (ref) | 1.04 (0.84, 1.29) 0.6928 | 1.36 (1.06, 1.76) 0.0191 | 1.71 (1.32, 2.22) 0.0001 |  |
| Yes | 1.0 (ref) | 0.89 (0.37, 2.15) 0.7968 | 0.87 (0.35, 2.17) 0.7710 | 0.87 (0.35, 2.17) 0.7710 |  |
| **CVD** |  |  |  |  | 0.3891 |
| No | 1.0 (ref) | 1.08 (0.88, 1.34) 0.4587 | 1.37 (1.06, 1.77) 0.0185 | 1.75 (1.34, 2.27) 0.0001 |  |
| Yes | 1.0 (ref) | 0.54 (0.23, 1.30) 0.1762 | 0.69 (0.27, 1.78) 0.4459 | 0.82 (0.34, 1.97) 0.6608 |  |

Note 1: The above model adjusted for age, race, education level, marital status, PIR, smoking status, alcohol consumption, diabetes, hypertension, CVD, and stroke. Note 2: In each case, the model was not adjusted for the stratification variable.
