# Supplementary material for: Association between cardiometabolic index and overactive bladder in adult American women: A cross-sectional study
Source: PLoS One. 2025 Jan 14;20(1):e0314594. doi: 10.1371/journal.pone.0314594 (PMC11731727; doi:10.1371/journal.pone.0314594)
Supplement: S4 Table — (DOCX) [file pone.0314594.s004.docx]

**Table S4.** Baseline table of participant population based on OAB subgroups.

| **Characteristics** | **OAB** | | ***P* value** |
| --- | --- | --- | --- |
|  | **No** | **Yes** |  |
| Number, n | 4394 | 1929 |  |
| Age (years, mean ± SD) | 46.15 ± 16.71 | 56.70 ± 15.45 | <0.0001 |
| Race (%) |  |  | <0.0001 |
| Mexican American | 7.97 | 7.48 |  |
| Other Hispanic | 5.87 | 5.04 |  |
| Non-Hispanic White | 67.77 | 69.29 |  |
| Non-Hispanic Black | 10.18 | 12.98 |  |
| Other Race | 8.2 | 5.22 |  |
| Education level (%) |  |  | <0.0001 |
| Less than high school | 12.4 | 20.87 |  |
| High school | 21.27 | 26.35 |  |
| More than high school | 66.33 | 52.78 |  |
| Marital status (%) |  |  | <0.0001 |
| Never married | 18.42 | 9.39 |  |
| Married/Living with partner | 62.16 | 57.04 |  |
| Widowed/divorced/Separated | 19.43 | 33.57 |  |
| PIR (%) |  |  | <0.0001 |
| <1.3 | 21.42 | 27.82 |  |
| 1.3 - 3.5 | 34.8 | 39.31 |  |
| ≥3.5 | 43.78 | 32.88 |  |
| BMI (%) |  |  | <0.0001 |
| <25 | 37.51 | 21.49 |  |
| 25 - 30 | 28.59 | 27.44 |  |
| ≥30 | 33.9 | 51.07 |  |
| Smoking status (%) |  |  | <0.0001 |
| Never | 63.51 | 55.75 |  |
| Now | 16.7 | 19.42 |  |
| Former | 19.79 | 24.83 |  |
| Alcohol intake (%) |  |  | <0.0001 |
| No | 10.26 | 15.53 |  |
| Yes | 89.74 | 84.47 |  |
| Hypertension (%) |  |  | <0.0001 |
| No | 69.16 | 42.04 |  |
| Yes | 30.84 | 57.96 |  |
| Diabetes (%) |  |  | <0.0001 |
| No | 90.61 | 75.35 |  |
| Yes | 9.39 | 24.65 |  |
| Stroke (%) |  |  | <0.0001 |
| No | 97.86 | 93.69 |  |
| Yes | 2.14 | 6.31 |  |
| CVD (%) |  |  | <0.0001 |
| No | 96.44 | 87.78 |  |
| Yes | 3.56 | 12.22 |  |
| TC (mmol/L, mean ± SD) | 5.05 ± 1.06 | 5.14 ± 1.09 | 0.0031 |
| HDL-C (mmol/L, mean ± SD) | 1.56 ± 0.44 | 1.49 ± 0.42 | <0.0001 |
| TG (mmol/L, mean ± SD) | 1.22 ± 1.01 | 1.44 ± 1.08 | <0.0001 |
| WHtR (mean ± SD) | 0.58 ± 0.10 | 0.64 ± 0.11 | <0.0001 |
| Height (cm, mean ± SD) | 162.42 ± 6.82 | 160.84 ± 6.99 | <0.0001 |
| Waist circumference (cm, mean ± SD) | 94.88 ± 16.07 | 103.21 ± 17.47 | <0.0001 |
| CMI (mean ± SD) | 0.57 ± 0.76 | 0.77 ± 1.11 | <0.0001 |

Continuous variables are expressed as mean and standard deviation (SD) and categorical variables are expressed as percentages.

Abbreviations: PIR, poverty income ratio; BMI, body mass index; HDL-C, high-density lipoprotein cholesterol; TG, triglyceride; TC, total cholesterol; CMI, cardiometabolic index; WHtR, waist-to-height ratio; WC, waist circumference; CVD, cardiovascular disease.
