# Supplementary figures and images for: Association between cardiometabolic index and overactive bladder in adult American women: A cross-sectional study
Source: PLoS One. 2025 Jan 14;20(1):e0314594. doi: 10.1371/journal.pone.0314594 (PMC11731727; doi:10.1371/journal.pone.0314594)

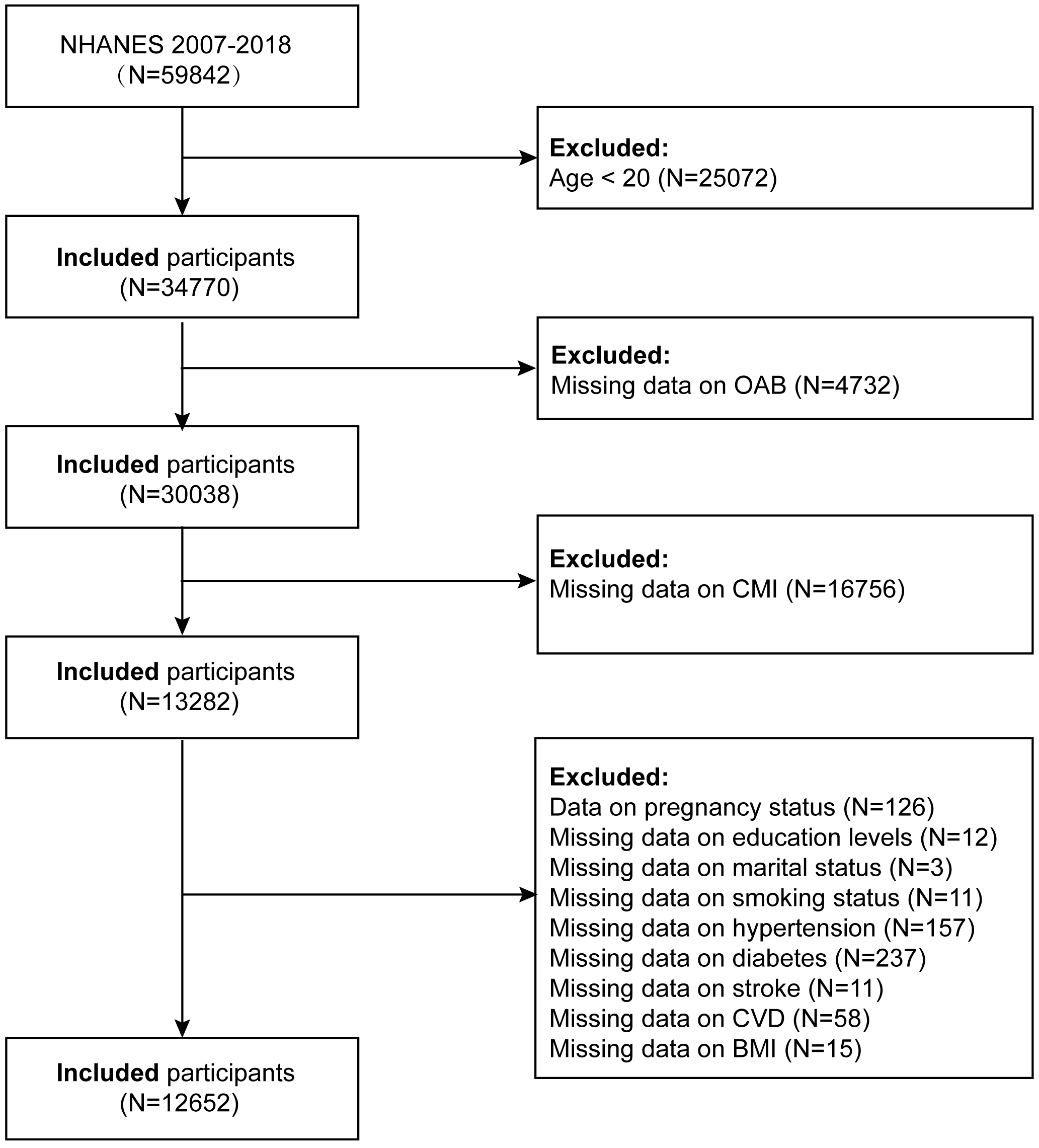

Supplement: S1 Fig — (TIF) [file pone.0314594.s005.tif]
